# Sex in Late Life: Assessing Adults’ Expectations and the Role of Healthcare Professionals

**DOI:** 10.1093/geroni/igaa057.1003

**Published:** 2020-12-16

**Authors:** Jill Naar, Raven Weaver, Shelbie Turner

**Affiliations:** 1 Appalachian State University, Boone, North Carolina, United States; 2 Washington State University, Pullman, Washington, United States; 3 Oregon State University, Corvallis, Oregon, United States

## Abstract

Sexual activity contributes to quality of life throughout the lifespan. However, stigma about sex in late life influences older adults’ perceptions and healthcare professionals’ perceptions of older adults’ sexual health/behaviors. Using a multi-methods approach, we examined attitudes and knowledge about sexual health/behaviors in late life. Using longitudinal data from the Midlife in the US Study (Wave 1-3; N=7049), we ran age-based growth curve models to analyze changes in levels of optimism about sex in their future. We also piloted a survey with healthcare professionals assessing attitudes, knowledge, and awareness of policy about sexual health/behaviors among older adults. Adults’ expectations became less optimistic with increased age (β = -0.1, SE = 0.003, p < .0001). Men were more optimistic than women at age 20 (p = 0.016), but men’s optimism decreased over the life course at a faster rate than did women’s (p < .0001), so that from ages 40-93, men were less optimistic than women. Among healthcare professionals (N=21), the majority indicated never or rarely asking their clients about sexual history or health/behaviors; however, they indicated some knowledge about issues relevant to older adults (e.g., safe-sex practices, sexual dysfunction). Few indicated awareness about policies related to sexual behavior among residents (i.e., issues of consent, STIs). Among adults, there is a need to address declining optimism for expectations about sex in late life. Health professionals are well-situated to raise awareness and normalize discussions about sexual health, thus countering negative stigma and contributing to increasing optimism for expectations to remain sexually active.

